# Rapid Diagnostic Centres and early cancer diagnosis

**DOI:** 10.3399/bjgp21X717413

**Published:** 2021-10-29

**Authors:** Simon Erridge, Georgios Lyratzopoulos, Cristina Renzi, Andrew Millar, Richard Lee

**Affiliations:** Epidemiology of Cancer Healthcare Outcomes (ECHO) Group, Department of Behavioural Science and Health, University College London, London.; ECHO Group, Department of Behavioural Science and Health, University College London, London.; ECHO Group, Department of Behavioural Science and Health, University College London, London.; North Middlesex University Hospital, London, UK; The Royal Marsden and The Institute for Cancer Research National Institute for Health Research (NIHR) Biomedical Research Centre (BRC), The Royal Marsden NHS Foundation Trust, London.

Early cancer diagnosis is a clinical and research priority of the UK government. Earlier cancer diagnosis should enable identification of cancers at an earlier stage, leading to improved outcomes.^1^ This must be balanced with the potential physical and psychological harms of over-investigation and over-diagnosis.

The ‘two-week wait’ (2WW) referral pathway represents the most common route to cancer diagnosis. However, only 39% of cancer diagnoses were made via 2WW pathways in 2017, while significant proportions of diagnoses are made via other outpatient clinics (32%) or emergency presentation (19%), representing potentially missed diagnostic opportunities.^2^

Approximately 50% of cancer patients present with non-specific but concerning symptoms of cancer (NSCS).^3^ Compared to ‘alarm symptoms’ these have low predictive values for cancer and are less indicative of site-specific disease; consequently, they are not reflected in 2WW referral criteria.^3^ These patients frequently are referred later for specialist investigation and have more advanced disease.^4^ A principal goal for the new NHS Rapid Diagnostic Centres (RDCs) is to provide a pathway for patients with NSCS to detect cancer earlier, where treatment outcomes are more favourable.^1^ NHS England aims to provide full population coverage with RDCs by 2024.^5^

## NSCS

Despite their low predictive value and association with multiple diseases, many NSCS are considered as characteristic warning signs of cancer. These include unexpected weight loss, malaise, unexplained pain, new dyspnoea, and persistently abnormal blood tests.^4^^,^^6^^,^^7^ Unexpected weight loss was the most common symptom seen within an RDC pilot (66%) between 2016 and 2018;^7^ however, it has a relatively low predictive value for cancer.^6^^,^^8^^,^^9^ While patients with weight loss in isolation may not warrant further investigation, considering other clinical variables may increase the likelihood that weight loss is suggestive of cancer.^8^

Primary care is the optimum environment to navigate uncertainty and manage risk in patients with NSCS. For patients with vague symptoms requiring secondary care input, however, the lack of a dedicated pathway previously made negotiating referral pathways challenging and complex. 2WW pathways are designed to investigate symptoms with high predictive values for single-site cancers. NSCS are less specific to individual cancer sites and investigation could require several specialist referrals contributing to diagnostic delay, negative patient experience, and increased costs.^3^

## RDCs AND CHALLENGES OF DELIVERY

**Figure 1.**
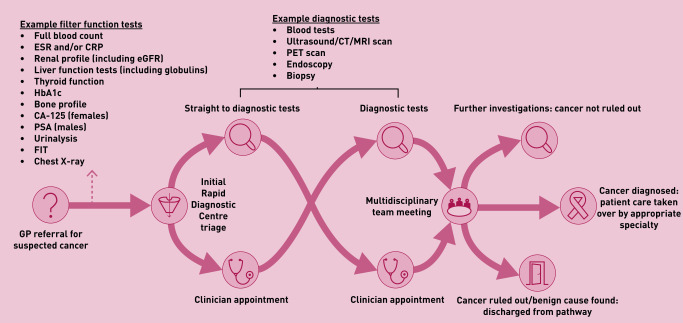
*Core pathway and referral set out within the NHS Implementation Specification.^5^ CA-125 = cancer antigen-125. CRP = C-reactive protein. CT = computed tomography. eGFR = estimated glomerular filtration rate. ESR = erythrocyte sedimentation rate. HbA1c = haemoglobin A1c. FIT = faecal immunochemical test. MRI = magnetic resonance imaging. PET = positron emission tomography. PSA = prostate specific antigen.*

RDCs are designed as a single point of access to multidisciplinary teams, supported by rapid diagnostics.^5^ NHS England, in concert with GPs from the RDC Expert Advisory Group, have outlined core referral criteria and pre-referral tests (Figure 1).^5^ These will continue to be refined iteratively in response to planned evaluation. Pre-referral testing is required to reduce time to diagnosis through guiding initial investigation strategy within RDCs, screening for non-cancer causes for symptoms, and ensuring patients are referred to site-specific cancer or non-cancer pathways if more appropriate. This helps ensure that the pathway manages the most appropriate cohort of patients to avoid unnecessary patient anxiety and over-investigation, while optimising cost-effectiveness.^10^ RDCs, which should comply with the Faster Diagnosis Standard, perform a diagnostic assessment and then refer patients to benign and cancer pathways or back to general practice as appropriate.^5^

General practice is integral to the success of the RDC pathway through early identification and timely referral of appropriate patients. This pathway hopefully empowers practitioners to refer patients early with NSCS or where there is otherwise strong clinical suspicion. Despite the low predictive values of individual symptoms, the development of RDCs represents a directive to target early diagnosis in those with the longest diagnostic intervals.^3^ This strategy has yielded promising conversion rates. The 2016–2018 RDC pilot had a conversion rate of 8%, with a benign diagnosis also provided for over 50% of patients.^7^ Four RDCs have since published their outcomes with conversion rates between 7% and 12%, exceeding the positive predictive value required of 2WW pathways.^6^^,^^9^^,^^11^^,^^12^

UK RDCs are based on a similar non-site-specific cancer pathway in Denmark, a country that has a similar healthcare system and focus on improving early diagnosis.^10^ Analyses of the Danish model have highlighted a conversion rate between 11% and 20%, with 22%–34% receiving diagnoses for benign conditions.^10^ However, higher conversion rates in Denmark may be secondary to the more liberal access to imaging prior to referral.^10^

The Port Talbot RDC conducted a cost-utility analysis that showed that seeing five patients per half-day session is associated with cost savings of £148.32 per patient and marginal improvements in quality of life compared to usual care.^12^ However, this study did not establish cost-effectiveness following discharge from RDCs.

While these findings are promising, and the rationale for RDCs is clear, it is not yet proven whether they will improve early diagnosis or cancer outcomes. Moreover, it is also important to assess whether patient experience is improved. Moving forward, data on Routes to Diagnosis should include RDCs to assess if they reduce the number of diagnoses made through emergency and outpatient routes.^2^

Currently there is geographical variation in access to RDCs. It is important, however, that patients who are unable to access RDCs due to local provisions still receive expeditious investigations. In this setting it would be prudent for Clinical Commissioning Groups to facilitate urgent direct-access investigations within primary care. Referrals to 2WW pathways should be carefully safety-netted to ensure continued investigation if no diagnosis is reached. Direct communication between GP and specialist, as well as parallel referrals, could be considered in select circumstances for such cases.

Prior to COVID-19, NHS England set a target of 20% of cancer patients with NSCS to be diagnosed within RDCs within the first year of deployment.^5^ This likely overstates the challenge within primary care to identify appropriate patients without adjunctive decision-making aids. This is particularly true of early-stage cancers, which may not evoke the same degree of clinical suspicion. The risks of delayed or missed cancer diagnosis must be balanced with those of over-investigation. The way in which this balance is struck is nuanced and cannot always be correct. Analysis of patient symptoms and outcomes from RDCs might help identify signatures that could be incorporated into clinical prediction tools to reduce uncertainty, reducing potential harms. RDCs also present an opportunity to design and evaluate novel diagnostics within a cohort of patients with an enhanced prevalence of cancer. Consequently, it is important that RDCs ensure they are research-ready to help meet the medium- and long-term goals of early diagnosis.

## CONCLUSION

Much of the skill of general practice is through navigating uncertainty with patients, no more so than in the setting of cancer and other serious conditions. RDCs have the potential to significantly improve early cancer diagnosis in the UK, through clinical activity and facilitating novel research. Input from primary care will be vital in determining the utilisation and effectiveness of the RDCs. However, there are several areas that still require research and development to support evolution of the RDC model:

1. What training and support is required for RDC clinicians? Will the role of diagnostician become a subspecialty for clinicians?
2. Can RDCs support better access to cross-sectional imaging and cancer diagnostics for primary care?
3. Which tests in primary care help achieve a diagnosis in RDCs, and which can be abandoned in favour of expediting referrals?
4. Can we develop better NSCS pathways supported by scoring systems and artificial intelligence, integrated with new cancer biomarkers?
